# Host–Pathogen Interactions: Organotypic Cultures to Unravel the Mysteries of the Primordial Hostility among Organisms

**DOI:** 10.3390/pathogens11030362

**Published:** 2022-03-16

**Authors:** Pasquale Marrazzo, Natalie Fischer, Claudia Nastasi, Monica Cricca, Daniela Fusco

**Affiliations:** 1 Department of Experimental, Diagnostic and Specialty Medicine, University of Bologna, 40126 Bologna, Italy; monica.cricca3@unibo.it; 2 Department of Infectious Diseases Epidemiology, Bernhard Nocht Institute for Tropical Medicine, 20359 Hamburg, Germany; natalie.fischer211@gmail.com; 3 Laboratory of Cancer Pharmacology, Department of Oncology, Istituto di Ricerche Farmacologiche Mario Negri IRCCS, 20156 Milan, Italy; claudia.nastasi@marionegri.it; 4 German Center for Infection Research (DZIF), Hamburg-Borstel-Lübeck-Riems, 20246 Hamburg, Germany

The interaction of humans with microorganisms represents a subtle balance between harm and good. Some microorganisms can act as pathogens for humans but not for other organisms [1], and the same microorganism can even be pathogenic for humans or not depending on its location in or on the body [2,3]. When microorganisms become pathogens, they clearly represent a threat to human life. Understanding host–pathogen interactions is the cornerstone for developing diagnostic tools as well as designing preventive and therapeutic proceedings [4]. Host–pathogen interactions are considered highly dynamic processes between diverse microbial pathogens and hosts in all stages of infection, from invasion to dissemination [5].

Immense breakthroughs have been achieved in the field of host–pathogen interaction, establishing the principles of eradication [6,7] and allowing for the control of several pathogens, thus leading to significant improvements in the life expectancy of humankind. Unfortunately, still a lot remains unsolved. In fact, the changing climate, together with massive urbanization and the intense global travelling behaviors of humans are quickly changing the plethora of pathogens [8] to which we are exposed. This translates to the need for continuous technological innovation, an ability to promptly identify them, and the ability to study interactions with their hosts.

The current COVID-19 pandemic has demonstrated how the use of innovative technologies such as mRNA vaccines [9] allow for approvals for new vaccines within 300 days [10]. This global experience has undoubtedly promoted the use of innovative technologies in the field of infectious diseases, which are life saviors during situations that require rapid response [11]. 

One of the main limitations to unravelling the complexities behind host–pathogen cross-talk is often represented by the lack of an appropriate model for studying the different stages of infection [12]. While scientists rely on animal models to study infection for a specific pathogen, for other pathogens, this is not always possible [13]. Moreover, animal models are costly and, in some cases, ethically debatable [14]. For these reasons, 3D in vitro tools represent an interesting alternative to minimizing or to entirely replacing animal use in pre-clinical studies.

The Special Issue “New Tools in 3D Host–Pathogen Interactions” has gathered six publications, including three original articles and three reviews, that clearly underline the need and the interest of the scientific community in increasing the use of 3D cell cultures, aiming to understand the intricate mechanisms behind host–pathogen interactions. 

*Shigella* is described by the World Health Organization (WHO) as the leading bacterial cause of diarrhea and the second-leading cause of diarrhea-associated mortality in 2016, especially in children < 5 years of age. It accounts for approximately 212,000 deaths and about 13% of all diarrhea- associated deaths worldwide [15,16]. Treatments for shigellosis are available and mostly consist of antibiotics that are not always accessible for the most highly affected populations, which are mainly located in low- and middle-income countries [17]. Moreover, the rising drug resistance to fluoroquinolones and cephalosporines [18], reducing the effectiveness of the treatment, places this genus of bacteria among pathogens of concern, with a need for the development of new antimicrobials and vaccines [15]. The lack of preclinical models for *Shigella* is concerningly delaying the identification of such new tools. Pilla et al. [19] showed how the use of an enteroid model can represent a useful tool in validating molecules of interest for the management of shigellosis. Their findings provide clear evidence that human enteroids are a relevant model for mimicking both invasion and intracellular replication of *Shigella sonnei* and, consequently, for the testing of vaccine candidates. 

Even though much remains to be carried out to promote 3D human cellular systems as routine models for studying host–pathogen interactions, promising advancements have been made in the field of human–staphylococcal interactions. *Staphylococcus aureus (S. aureus)* is a Gram-positive bacteria, part of the human nose and skin microbiota, but it also causes a wide variety of clinical manifestations both in the community and in healthcare facilities [20]. Since the 1960s, methicillin-resistant *S. aureus* (MRSA) has emerged [21], posing a worldwide threat in terms of antimicrobial resistance (AMR). In 2019, MRSA has been included as a new AMR indicator in the Sustainable Developmental Goals (SDGs) monitoring framework [22]. Chronic and prolonged infections with *S. aureus* represent the highest burden of disease, with decreasing likelihood of treatment success as the infection becomes established [23]. In this Special Issue, two different publications [24,25] showed the potential of 3D models in gaining more insight towards controlling the burden caused by such a threatening pathogen. Hofstee et al. [24] showed that staphylococcal abscess communities cause myeloid-derived suppressor cell (MDSC) expansion from bone marrow cells and identified possible mediators to be targeted as an additional strategy for treating chronic *S. aureus* infections. Parente et al. [25] developed a 3D model of osteomyelitis (OM), a bone condition primarily induced by *S. aureus* infections, based on co-cultures of *S. aureus* and murine osteoblastic MC3T3-E1 cells on magnesium-doped hydroxyapatite/collagen I (MgHA/Col) scaffolds that closely recapitulate the bone extracellular matrix. This model shows great potential for the study of OM caused by *S. aureus* infections. 

The Zika virus (ZIKV) has received particular attention in the past years, following the big epidemics reported in south America in 2016 [26]. The clinical relevance of ZIKV is mostly connected to fetal or early life infections that can lead to neurological disorders that severely impact the quality of life and life expectancy of affected individuals [27]. Additionally, in this case, the lack of appropriate animal models heavily limits progress towards unraveling host–pathogen interaction mechanisms for the identification of effective vaccines. In their review, Marrazzo et al. [28] discussed the use of brain organoids as valuable models for ZIKV infection, underlining the advantages of these models in accelerating research. As described by Hopkins et al. [29], brain organoids represent invaluable models in studying several pathogenic disorders that affect the brain and the nervous system. In their review, they thoroughly discussed the effectiveness and advantages of the use of these models and recognized the existing cost and technology limitations. Nevertheless, these two examples clearly highlight the call for more investments to improve the technology in this field, since the outcomes promise to be invaluable in terms of scientific findings. 

Finally, Baddal and Marrazzo [30] described different microfluidic human tissue models by taking advantage of modeling pathophysiology in a dynamic 3D host–pathogen microenvironment. In particular, they reviewed organ-on-chip technology as a tool for refining organ-specific responses during infection, such as in the lung, intestine, kidney, and blood–brain barrier.

In conclusion, we believe that this Special Issue illustrates the potential of advanced in vitro models based on 3D cell cultures as significant tools useful in improving knowledge about host–pathogen interactions. At the same time, it clearly shows how limited the use of these tools still is. We hope that this Special Issue contributes to widening the awareness and stimulating researchers to further develop and use 3D tools in the field of host–pathogen interaction.
